# Flexible Sensing Electrodes: From Materials Design to Device Fabrication

**DOI:** 10.3390/ma19143036

**Published:** 2026-07-14

**Authors:** Haiyi Zhang, Yitao Wang, Xueming Duan, Mengbo Zhang, Lijing Han, Sakil Mahmud, Ruoyu Zhang

**Affiliations:** 1 College of Material Science and Chemical Engineering, Ningbo University of Technology, Ningbo 315211, China; zhanghaiyi@nbut.edu.cn (H.Z.); wangyitao@nbut.edu.cn (Y.W.); duanxueming@nbut.edu.cn (X.D.); zhangmengbo@nbut.edu.cn (M.Z.); 2 Ivory V. Nelson Center for the Sciences, Department of Chemistry and Physics, Lincoln University, 1570 Baltimore Pike, Oxford, PA 19352, USA; 3 Department of Textile Engineering, Faculty of Engineering, Daffodil International University, Dhaka 1216, Bangladesh

**Keywords:** flexible sensing electrode, wearable device, electrophysiological monitoring, electronic skin

## Abstract

Driven by the rapid advancement of routine health management and intelligent human–machine interaction technologies in the post-pandemic era, wearable flexible sensing electrodes have emerged as key enabling platforms for long-term physiological monitoring and next-generation bioelectronic systems. Compared with conventional rigid electrodes, flexible sensing electrodes offer superior mechanical compliance, lightweight characteristics, and improved biocompatibility, enabling stable acquisition of physiological signals under dynamic conditions. However, achieving the simultaneous optimization of conductivity, mechanical flexibility, biocompatibility, and long-term operational stability remains a significant challenge, requiring synergistic advances in material design and fabrication strategies. This review systematically summarizes recent progress in flexible sensing electrodes from three perspectives: material systems, fabrication technologies, and practical applications. Specifically, the intrinsic properties and design strategies of flexible substrates, sensing layers, and encapsulation layers are critically analyzed. Diverse fabrication approaches, including printing technologies, solution processing, photolithography, and laser-assisted manufacturing, are further discussed, with emphasis on scalability and engineering challenges. Finally, representative applications, current limitations, and future opportunities for intelligent, multimodal, and scalable flexible sensing systems are highlighted to guide future research and commercialization.

## 1. Introduction

In the post-pandemic era, the limitations of traditional disease-centered healthcare paradigms have become increasingly evident, particularly in addressing the growing demand for proactive and personalized health management. In response, routine health management has emerged as an alternative framework, emphasizing long-term monitoring and intervention of physiological status through continuous, non-invasive, and portable technologies. Within this paradigm shift, external bioelectronic interfaces, especially wearable electrodes, have become essential enabling platforms for high-fidelity physiological signal acquisition. Conventional rigid electrodes suffer from intrinsic limitations, including poor mechanical compliance, susceptibility to motion artifacts, limited long-term stability, and inadequate user comfort, restricting their applicability in emerging healthcare and human–machine interaction systems [1,2,3,4]. By contrast, wearable flexible electrodes, owing to their lightweight, superior biocompatibility, and mechanical deformability, can conform closely to dynamic biological surfaces, enabling stable, high-quality bioelectrical signal acquisition without significantly interfering with daily human activities.

An ideal wearable flexible sensing electrode should simultaneously exhibit high electrical conductivity, excellent mechanical flexibility and stretchability, favorable biocompatibility, strong skin affinity, and long-term operational reliability. However, achieving the simultaneous optimization of these often conflicting properties remains a major challenge [5,6]. Addressing this issue requires extensive interdisciplinary integration across materials science, micro- and nanofabrication, mechanics, bioengineering, and device physics. Recent advances arising from cross-disciplinary research have substantially accelerated the development of flexible sensing systems and enabled significant breakthroughs in device performance and functionality [7,8,9].

**Figure 1:**
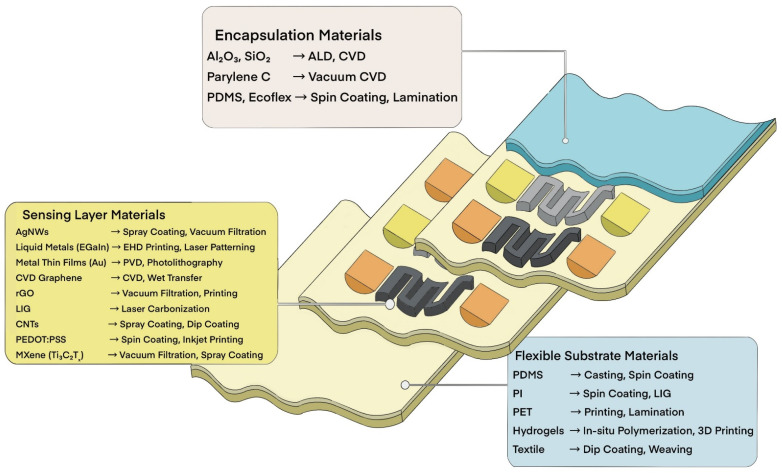
Materials of each layer in the flexible sensing electrode and their corresponding fabrication methods.

From a materials perspective, wearable flexible sensing electrodes generally adopt multilayer composite architectures composed of a flexible substrate, a conductive sensing layer, and a protective encapsulation layer [10] (Figure 1). The synergistic interactions among these functional components largely determine the overall device performance. The flexible substrate acts as the mechanical support and should maintain structural integrity while preserving flexibility and user comfort. Representative substrate materials include polymeric elastomers such as polydimethylsiloxane (PDMS) [11,12], polyimide (PI) [13,14], and polyethylene terephthalate (PET) [15], which possess excellent film-forming capability and tunable mechanical properties. In addition, hydrogels, owing to their tissue-like mechanical properties and favorable biocompatibility, have attracted considerable interest in constructing biomimetic interfaces [16,17], whereas textile-based substrates, with their intrinsic porosity and breathability, are particularly advantageous for long-term wearable integration [18]. The sensing layer serves as the central functional unit responsible for signal transduction and conversion, and the choice of material critically determines sensing sensitivity and selectivity. It should be noted that most polymers are intrinsically insulating (conductivity <10^−10^ S/cm) and require either incorporation of conductive fillers [19,20], use of intrinsic conductive polymers such as PEDOT:PSS [21,22] and PPy [23,24], or ionic/protonic acid doping [25] to achieve electrical functionality. Current conductive materials can generally be classified into four categories: metal-based materials, e.g., silver nanowires (AgNWs) and liquid metals, which possess exceptionally high intrinsic conductivity but face challenges in patterning precision and long-term stability [19,26]; carbon-based materials, such as graphene and carbon nanotubes (CNTs), characterized by high chemical stability and large specific surface area suitable for constructing highly sensitive interfaces [20,27]; conductive polymers [poly(3,4-ethylenedioxythiophene): poly(styrenesulfonate) represented by PEDOT:PSS], which combine flexibility with excellent solution processability but still suffer from environmental instability [21,28]; and emerging transition-metal carbides/nitrides (MXenes), which exhibit metallic conductivity and hydrophilic surfaces and have shown significant potential in flexible electrochemical sensing applications [22,29].

Fabrication methodology serves as the critical bridge linking material selection with the ultimate performance of flexible electrodes. The chosen fabrication route directly influences electrical conductivity, mechanical robustness, permeability, biocompatibility, and production cost. Existing fabrication approaches encompass the complete process chain from material synthesis and deposition to patterning and final device assembly, and can generally be categorized into deposition-based methods, printing technologies, solution processing, photolithography, and laser-assisted techniques. Deposition approaches offer operational simplicity and cost-effectiveness for large-area film fabrication but often exhibit limitations in structural precision and pattern controllability. Printing technologies, benefiting from digital design capability, mask-free processing, and high material utilization efficiency, have demonstrated substantial promise in rapid prototyping and customized manufacturing [17,30]. Solution-processing methods, including spin coating, drop casting, and dip coating, enable uniform thin-film preparation and compatibility with diverse material systems, although precise multilayer integration remains challenging [31,32]. Photolithography offers unmatched advantages in micro- and nanoscale patterning due to its high resolution and mature industrial infrastructure, yet its processing complexity and stringent conditions limit its compatibility with flexible substrates [31,32,33,34,35]. More recently, laser processing technologies have emerged as powerful non-contact, maskless strategies, creating new opportunities for high-throughput, high-precision manufacturing of flexible electrodes.

In summary, rational material selection and fabrication optimization are the primary drivers of advances in wearable flexible electrode technologies. Despite substantial progress in recent years, considerable challenges remain in achieving flexible electrodes with simultaneously high performance, low manufacturing cost, and scalable production capability. This review systematically summarizes recent advances in flexible sensing electrodes from the perspectives of material systems, fabrication strategies, and representative applications. Particular emphasis is placed on elucidating the underlying mechanisms by which different design strategies enhance device performance while critically discussing current technological bottlenecks and future research directions. Through this analysis, we aim to provide a comprehensive framework to guide the continued development and eventual commercialization of next-generation flexible sensing electrodes.

## 2. Material System of Flexible Sensing Electrodes

The structural architecture of flexible sensing electrodes generally comprises four key components: a flexible substrate, a sensing layer, a functional layer, and an encapsulation layer. The flexible substrate provides mechanical support and deformability, while parameters including Young’s modulus, elongation at break, and surface energy directly govern device compliance and wearer comfort; the sensing layer is responsible for electrical signal transmission and transduction and serves as the central functional component of the electrode system; the functional layer refers to specialized active materials that provide targeted sensing capabilities, such as piezoelectric, triboelectric, thermoelectric, or thermoresponsive layers, which operate synergistically with the sensing layer to detect physical or biochemical stimuli; the encapsulation layer critically influences device stability, environmental tolerance, and operational lifetime. Based on transduction mechanisms, flexible sensors can be classified into resistive, capacitive, piezoelectric, triboelectric, thermoelectric, and other categories [36]. The diverse operating principles and application environments of these devices impose distinct requirements on conductivity, sensitivity, dynamic response range, and environmental stability, thereby necessitating a highly diversified material selection strategy.

### 2.1. Flexible Substrate Materials

Substrate materials are fundamental components of flexible sensing systems, providing mechanical support and enabling deformability for both sensing and functional layers. Their intrinsic properties directly influence key device characteristics, including flexibility, stretchability, long-term stability, breathability, and biocompatibility. An ideal flexible substrate should possess a low elastic modulus, high elongation at break (>100%), excellent resilience, and efficient moisture permeability while exhibiting mechanical characteristics comparable to human skin (Young’s modulus approximately 0.1–1 MPa), thereby ensuring long-term wearing comfort and stable physiological signal acquisition [37,38].

#### 2.1.1. Polymer-Based Substrate Materials

Polymeric elastomers can generally be categorized into physically and chemically cross-linked systems, offering broad tunability of physicochemical properties and functional performance. Owing to their excellent elastic recovery and fatigue resistance, these materials have become among the most widely adopted substrate candidates for flexible sensing devices.

**Figure 2:**
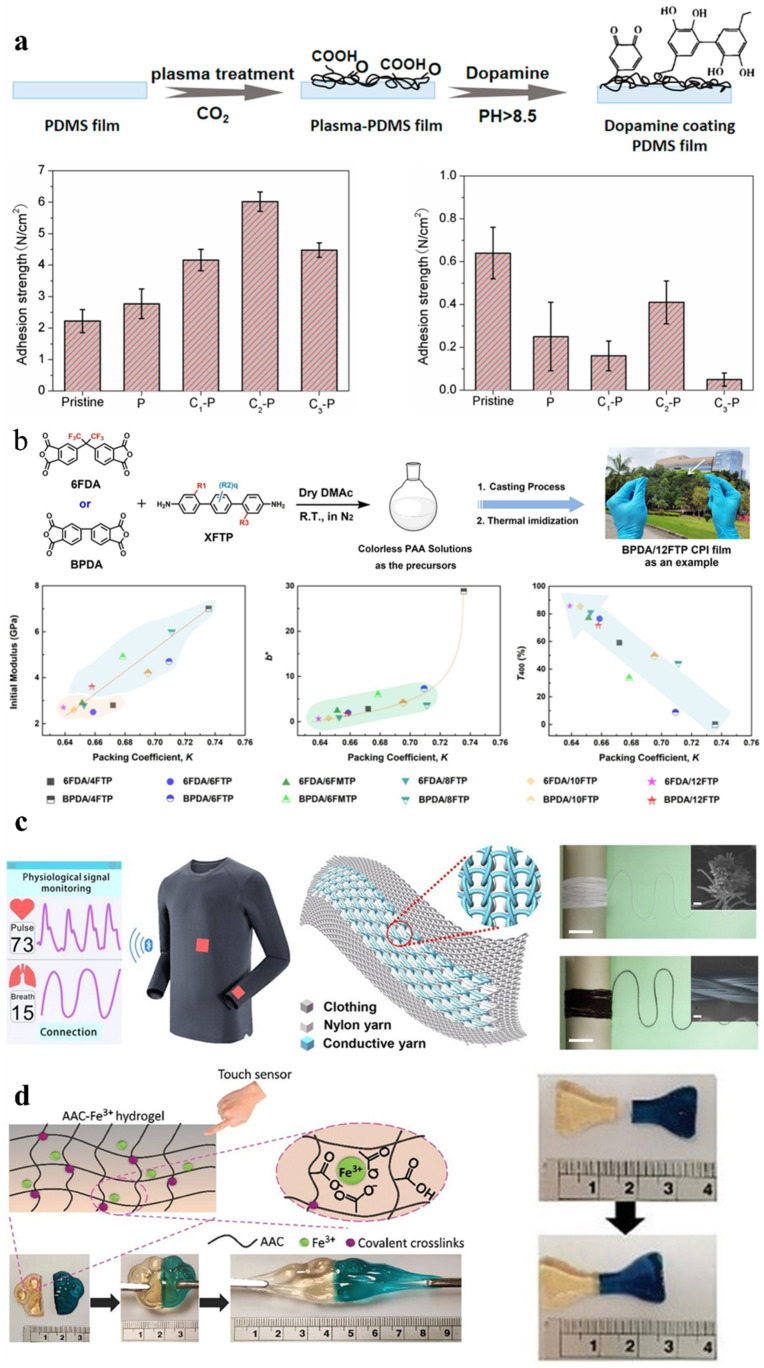
(**a**) Experimental process of plasma-assisted dopamine modification of PDMS and the shear and normal adhesion strengths at different ratios [41]. (**b**) Polymerization and casting process of colorless PI (CPI) films, and the initial modulus, b*, and T_400_ transmittance of materials with different compositions [42]. (**c**) Integration of triboelectric all-textile sensor arrays (TATSAs) into a shirt for real-time monitoring of pulse and respiration, including a magnified view of the sensor, a photograph of the conductive yarn, and a cross-sectional SEM image [43]. (**d**) Concept and self-healing process of the crosslinked acrylic acid (AAC)–Fe^3+^ hydrogel [44].

PDMS is among the most widely used elastomeric substrates because of its high elongation at break (>150%), excellent biocompatibility, high optical transparency (visible-light transmittance >90%), and superior chemical stability. PDMS can be readily processed into diverse geometries via spin coating, casting, and molding, while surface plasma treatment or chemical modification can enhance interfacial compatibility with functional materials. However, its intrinsically low moisture permeability, with a water vapor transmission rate (WVTR) of approximately 10^−15^ g·m/m^2^·Pa·s, limits its suitability for long-term wearable applications. Furthermore, its low surface energy (~20 mN/m) and significant chemical disparity between silicon- and carbon-based backbone structures result in weak interfacial adhesion with many functional materials, thereby limiting the strain-sensing range and signal stability [39]. Recent strategies, including porous structural engineering, incorporation of hygroscopic fillers, and copolymerization or blending with hydrophilic polymers, have substantially improved breathability and interfacial adhesion [40,41] (Figure 2a). Nevertheless, insufficient bonding strength between PDMS and sensing layers, as well as vulnerability to certain organic solvents, remain unresolved challenges [39].

Polyurethane (PU) represents another important elastomeric substrate. Its molecular structure comprises thermodynamically incompatible hard and soft segments that form microphase-separated morphologies, thereby endowing the material with excellent elasticity, abrasion resistance, and tear strength. Owing to the large design flexibility afforded by various diisocyanates, polyols, chain extenders, and tunable hard-to-soft segment ratios, PU exhibits a remarkably broad range of mechanical properties, with Young’s modulus spanning from several kilopascals to hundreds of megapascals. Such tunability provides substantial advantages for wearable systems requiring high resilience and fatigue resistance [45,46,47]. In addition, thermoplastic PU (TPU) and waterborne PU (WPU) are compatible with injection molding, extrusion, and solution-processing techniques, facilitating scalable manufacturing. Moreover, the recyclability of TPU provides additional benefits for sustainable flexible electronic systems [46,48].

Thermoplastic elastomers (TPEs) are processable materials whose physically cross-linked networks reversibly dissociate upon heating and reform during cooling. Owing to their compatibility with conventional polymer processing techniques such as injection molding and extrusion, TPEs are highly suitable for large-scale manufacturing and are often more cost-effective than PDMS and PU [49]. Their mechanical properties can be precisely tailored through block-composition engineering, enabling widespread applications in epidermal electronics and wearable sensing platforms [50].

PI is a high-performance polymer film characterized by excellent dimensional stability (glass transition temperature, T_g_ > 300 °C), robust mechanical strength (tensile strength > 150 MPa), and superior chemical resistance. These characteristics make PI highly suitable for flexible electronic systems that require high-temperature fabrication processes, such as photolithography and thermal annealing [51,52]. However, poor air permeability, limited degradability, and its characteristic yellow coloration constrain applications involving long-term wearability or optical transparency. Recent studies have introduced fluorinated monomers and flexible ether linkages to develop modified PI materials with reduced dielectric constants and enhanced optical transparency, thereby expanding their applicability in high-frequency flexible electronics [42,53] (Figure 2b).

PET and polyethylene naphthalate (PEN) are two widely used polyester materials. Due to their stable physicochemical properties, excellent optical transparency, and mature fabrication technologies, they are commonly used as substrates for flexible displays and optoelectronic sensing devices. PET films are lower-cost but exhibit relatively limited thermal stability, whereas PEN offers improved operating-temperature tolerance and barrier performance at a higher material cost [54,55].

Additional substrate candidates include high-performance polymers such as polyether ether ketone (PEEK). Owing to their excellent biocompatibility and chemical resistance, PEEK-based films have attracted considerable attention for implantable biomedical devices. However, their high production cost and relatively demanding processing conditions continue to restrict large-scale implementation [56,57,58].

From an environmental sustainability perspective, PDMS and other conventional elastomers pose significant end-of-life disposal challenges owing to their non-biodegradability and petrochemical-derived origins [39]. Amoah et al. (2025) addressed this by developing a chitosan/sorbitol substrate with a self-doped conductive polymer (Poly-A), in which >95 wt% of components are renewable and biodegradable, achieving 0.08 MΩ resistance at 0.15 wt% loading, >200% elongation, and 1.39 MPa Young’s modulus matching human skin [37,38], at substantially lower material cost [59].

#### 2.1.2. Hydrogel-Based Substrate Materials

Hydrogels are soft, hydrated materials composed of cross-linked polymer networks that contain substantial amounts of water. Their mechanical characteristics closely resemble those of biological soft tissues such as skin and muscle, with Young’s modulus typically ranging from 1 to 100 kPa, and they simultaneously exhibit favorable biocompatibility. Consequently, hydrogels have attracted considerable attention as substrate materials for flexible sensing systems. Their high water content (>70%) provides excellent ionic conductivity and superior biointerfacial compatibility.

Conductive hydrogels integrate electrical conductivity with the intrinsic softness of hydrogel systems, enabling their direct use as flexible sensing electrodes. Conductivity within conductive hydrogels generally arises from three mechanisms: incorporation of conductive polymers [e.g., PEDOT:PSS and polypyrrole (PPy)] that establish electronic transport through π-conjugated frameworks [60]; incorporation of conductive fillers such as CNTs, graphene, and MXenes that create percolative conductive pathways [61]; and incorporation of ionic conductive media including salt solutions or ionic liquids, where signal transfer occurs via ion migration [62]. Conductive hydrogels have demonstrated distinct advantages in strain sensing, pressure sensing, and electrophysiological signal acquisition, owing to their low interfacial impedance (<10^4^ Ω·cm^2^), which substantially improves signal quality.

Self-healing hydrogels represent one of the most active frontiers in hydrogel research. By incorporating dynamic covalent interactions, including disulfide, borate ester, and acylhydrazone bonds, or reversible noncovalent interactions such as hydrogen bonding, metal coordination, and host–guest interactions, these materials can autonomously restore structural integrity and functionality following damage. Integration of self-healing hydrogels into flexible sensing systems can significantly enhance device durability and operational lifetime, particularly for wearable applications exposed to repeated deformation and mechanical stress [44] (Figure 2d).

#### 2.1.3. Fabric-Based Substrate Materials

Textile substrates possess inherent porous architectures and excellent breathability, with WVTRs reported to be two to three orders of magnitude higher than those of PDMS, making them particularly suitable for long-term wearable applications. Fibers and fabrics provide excellent comfort and mechanical flexibility, while sensing functionality can be introduced by integrating conductive materials. Common fabrication approaches include conductive fiber weaving, conductive coatings, and in situ polymerization techniques. Natural fibers such as cotton, silk, and wool exhibit favorable biocompatibility and moisture absorption, whereas synthetic fibers, including polyester and nylon, provide superior mechanical strength and durability. More recently, knitted textile sensors have attracted considerable attention for healthcare and motion-monitoring applications because of their high extensibility (>200%) and excellent skin conformity [63,64] (Figure 2c).

### 2.2. Sensing Layer Materials

The sensing layer represents the central functional component of flexible sensing systems, enabling signal detection, transduction, and transmission. Its physicochemical characteristics directly determine key sensing metrics, including sensitivity, response time, detection limit, and signal-to-noise ratio. In addition to these established material systems, binary transition-metal oxides (e.g., NiMoO_4_, CoMoO_4_) and binary metal oxides (e.g., BaMoO_4_, CaMoO_4_) have recently been investigated as electrochemically active materials, leveraging synergistic multi-cation redox activity to achieve specific capacitance exceeding 450 F/g. However, their intrinsic brittleness (fracture strain < 1%) and high-temperature synthesis requirements (>400 °C) currently preclude their integration with flexible polymer substrates, confining them to rigid-platform laboratory studies [65]. Based on intrinsic material properties, conductive materials used in sensing layers can generally be categorized into four major classes: metal-based materials, carbon-based materials, conductive polymers, and emerging two-dimensional (2D) materials such as MXenes. However, materials with high intrinsic conductivity often exhibit insufficient flexibility and processability, necessitating blending, doping, or hybridization with flexible polymer matrices to construct mechanically compliant and functional sensing architectures. Table 1 systematically compares the major sensing-layer materials in terms of conductivity, stretchability, biocompatibility, environmental stability, and cost, providing a practical framework for material selection across different application scenarios.

#### 2.2.1. Metal-Based Conductive Materials

**Figure 3:**
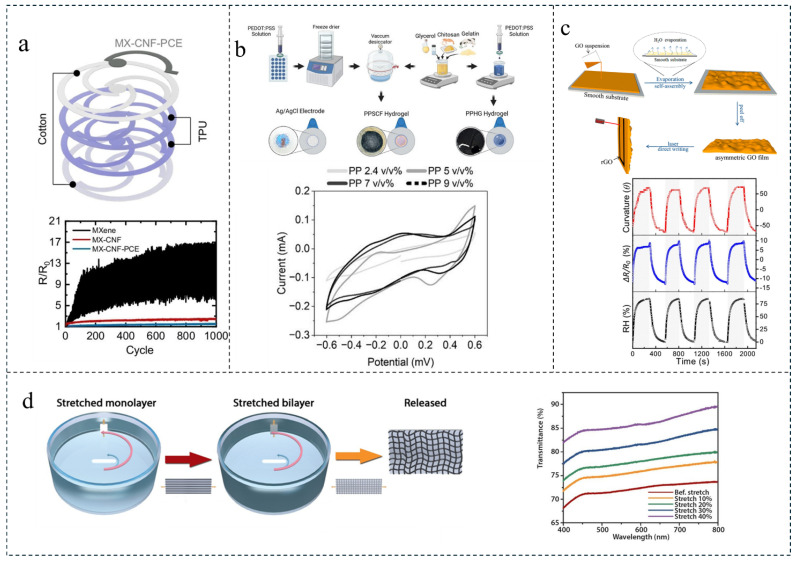
(**a**) Structural layout of FLEXER and its performance under 1000 cycles of 15% bending strain test [90]. (**b**) One-pot synthesis of the hydrogel substrate and the processing routes to form PEDOT:PSS scaffold (PPSCF) and PPHG variants, with the cyclic voltammetry tests of the electrodes shown below [23]. (**c**) Preparation scheme of the GO film responsive actuator, and the time-dependent curves of film curvature (θ, red line), relative resistance change of the embedded rGO sensor (ΔR/R_0_, blue line), and relative humidity variation (black line) [73]. (**d**) Left: Schematic of the layer-by-layer stirring-assisted alignment. The AgNW film exhibits a cross-aligned nanostructure in the stretched state and an entangled-oriented structure in the released state. Right: Transmittance of the 10 min samples under different applied strains [67].

Metal-based conductive materials occupy a central position in flexible electrode technologies because of their exceptionally high intrinsic electrical conductivity (silver: 6.3 × 10^7^ S/m; copper: 5.8 × 10^7^ S/m). Representative systems include AgNWs, silver nanoparticles (AgNPs), liquid metals, and metallic thin films. Among them, AgNWs have become a dominant choice for transparent, flexible electrodes because of their high aspect ratio (>1000) and excellent optoelectronic properties. Conductivity in AgNW networks arises from percolation pathways, and the trade-off between optical transmittance and sheet resistance can be optimized by precisely controlling nanowire density and length distribution. However, contact resistance at AgNW junctions remains a major factor limiting network conductivity and is commonly reduced through post-treatment approaches such as thermal pressing, plasma processing, or electrical welding. Additional challenges include electrochemical corrosion, atmospheric sulfidation of silver, and weak adhesion between AgNWs and polymer substrates. Recent studies have demonstrated that coating AgNWs with ultrathin graphene layers or metal oxides (e.g., ZnO and TiO_2_) effectively suppresses oxidation and improves interfacial bonding. Nevertheless, these additional processing steps inevitably increase fabrication complexity and production cost [66,67] (Figure 3d).

Gallium-based liquid metals, including eutectic gallium-indium (EGaIn) and gallium-indium-tin alloys, are considered ideal materials for highly stretchable electrodes because they combine metallic conductivity (~3.4 × 10^6^ S/m) with room-temperature fluidity (viscosity ~2 mPa·s). A unique feature of liquid metals is their ability to maintain continuous conductive pathways under tensile strains exceeding 100%, thereby preventing conductive network failure commonly observed in conventional rigid conductive fillers under large deformation [68]. In terms of high-resolution fabrication, a constrained-template electrohydrodynamic (EHD) printing strategy reported in 2024 reduced the linewidth of EGaIn electrodes to approximately 20 μm while preserving ~100% stretchability and excellent mechanical durability over 10,000 deformation cycles, achieving patterning resolutions comparable to conventional photolithography [77]. In addition, laser-assisted technologies have opened new opportunities for mask-free fabrication of liquid metal electrodes. Recent studies have reported ultraviolet (UV)-pulsed laser-assisted direct patterning of EGaIn electrodes and femtosecond-laser-enabled herringbone-selective dewetting approaches, providing promising pathways toward high-precision manufacturing [78,79].

#### 2.2.2. Carbon-Based Conductive Materials

Carbon-based materials, including graphene, CNTs, carbon black, and carbon fibers, play an important role in flexible sensing systems due to their outstanding chemical stability, high specific surface area (>500 m^2^/g), favorable biocompatibility, abundant availability, and relatively low cost. These characteristics make them among the most promising material families for achieving scalable and economically viable flexible sensing platforms.

Graphene is a 2D material composed of a single atomic layer of sp^2^-hybridized carbon atoms and possesses exceptionally high carrier mobility (>200,000 cm^2^/V·s) and remarkable mechanical properties (Young’s modulus ~1 TPa; fracture strength ~130 GPa). Chemical vapor deposition (CVD) remains the standard method for producing high-quality, large-area graphene, typically involving graphene growth on copper substrates at approximately 1000 °C under H_2_/CH_4_ atmospheres [70]. However, transferring CVD-grown graphene onto flexible substrates often introduces wrinkles, cracks, and contamination, compromising device performance. Although reduced graphene oxide (rGO) exhibits lower conductivity (~10^2^–10^4^ S/m) than CVD graphene, it can be readily produced from solution-processable graphene oxide (GO) via chemical, thermal, or electrochemical reduction routes. The simplicity and cost-effectiveness of this process make rGO suitable for large-area coating and printing. More recently, laser reduction of GO has attracted substantial interest because of its one-step, mask-free, and patternable fabrication capability [71,72] (Figure 3c).

CNTs are one-dimensional tubular nanostructures formed by rolling graphene sheets and can be classified as single-walled (SWCNTs) or multi-walled (MWCNTs). In flexible electrodes, CNTs are typically deposited onto deformable substrates through vacuum filtration, spray coating, or dip coating to establish percolative conductive networks. SWCNTs exhibit higher intrinsic conductivity and lower defect density, although challenges associated with dispersion and scalable low-cost production remain unresolved. MWCNTs are relatively inexpensive and easier to process, but their electrical conductivity is generally lower than that of SWCNTs [73,74]. Hybridization among carbon materials and between carbon materials and other conductive systems is an effective strategy for enhancing synergistic performance. For example, graphene/CNT hybrid films use graphene sheets as large-area conductive pathways, while CNTs bridge adjacent layers to form a three-dimensional (3D) “plane-line” conductive architecture, thereby substantially improving conductivity stability and structural uniformity [80,81,82]. Carbon black and carbon fibers, despite their relatively low conductivity, retain substantial practical value for disposable and low-cost sensing systems because of their economic advantages. Recent advances in surface functionalization and elastomeric composite engineering have significantly improved the sensitivity and linearity of carbon-black-based systems [83].

#### 2.2.3. Conductive Polymers

PEDOT:PSS is among the most widely used conductive polymers in flexible bioelectronics. PEDOT:PSS consists of electrically conductive PEDOT chains and insulating PSS counterion chains, exhibiting excellent film-forming capability, favorable optical transparency, and tunable conductivity that can be enhanced from approximately 10^−3^ S/cm to values exceeding 1000 S/cm through secondary doping strategies.

The advantages of PEDOT:PSS as a sensing material stem from several distinct characteristics. First, its mechanical and electrochemical compatibility with biological tissues significantly surpasses that of conventional metallic electrodes. PEDOT: PSS exhibits a Young’s modulus in the tens of megapascals range, whereas metals typically possess moduli in the gigapascal range. This improved modulus matching reduces mechanical stress at the tissue-electrode interface and consequently minimizes inflammatory responses. Second, PEDOT:PSS functions as a mixed ionic-electronic conductor capable of simultaneously supporting ion and electron transport. This property substantially decreases electrode-tissue interfacial impedance and improves signal acquisition quality, particularly for low-frequency electrophysiological signals such as electrocardiography (ECG) and electroencephalography (EEG) [84,85].

Alsaafeen et al. (2025) [23] reported a scalable one-pot fabrication strategy for a PEDOT:PSS electrode exhibiting intrinsic stretchability, self-adhesion, and biocompatibility. In a cohort study involving 39 participants, PEDOT:PSS electrodes outperformed conventional Ag/AgCl electrodes across multiple electrophysiological modalities. ECG recordings exhibited reduced motion artifacts, improved signal-to-noise ratios, and clearer preservation of diagnostic features; EEG recordings demonstrated enhanced α-δ rhythm separation; and EMG signals showed increased amplitudes and improved signal clarity. Moreover, machine learning-assisted analysis revealed a 2.2-fold increase in inter-lead ECG classification accuracy. The inclusion of multimodal electrophysiological validation in a moderately sized human cohort provides strong evidence supporting the translational potential of PEDOT:PSS electrodes for wearable and clinical monitoring applications [86].

Polyaniline (PANI) and PPy are two additional conductive polymers that have been extensively investigated. PPy can be synthesized through chemical oxidative polymerization or electrochemical deposition and exhibits excellent electrochemical activity and biocompatibility, making it particularly suitable for biosensing and neural electrode coatings [23,24]. PANI exhibits reversible transitions between insulating and conductive states through protonic acid doping and has therefore attracted considerable attention in pH and gas sensing applications. However, compared with PEDOT:PSS, both PPy and PANI exhibit inferior chemical stability, particularly under repeated oxidation-reduction cycling, and limited solution processability, thereby restricting their applicability in long-term implantable systems [25,87] (Figure 3b).

Furthermore, fundamental studies on charge transport in doped organic semiconductors have demonstrated that recombination kinetics (Langevin versus non-Langevin regimes) profoundly influence the carrier concentration and Hall voltage, providing theoretical guidance for optimizing doping strategies in conductive polymer sensing layers [88].

#### 2.2.4. MXene

MXenes constitute a family of 2D transition metal carbides, nitrides, and carbonitrides obtained through selective etching of the A-layer atoms from MAX-phase precursors. The most extensively investigated example, Ti_3_C_2_T_x_, was first reported by Gogotsi and co-workers in 2011, establishing an entirely new branch of 2D materials. MXenes generally follow the formula M_n+1_X_n_T_x_, where M represents a transition metal, X denotes carbon and/or nitrogen, and T_x_ corresponds to surface terminations such as –OH, –O, and –F [75].

MXenes exhibit several properties that are highly advantageous for flexible electrode applications, including metallic-grade conductivity (10,000–20,000 S/m), excellent hydrophilicity and solution processability, high specific surface area, substantial electrochemical activity, and mechanical flexibility. The bending stiffness of monolayer Ti_3_C_2_T_x_ is lower than that of both graphene and MoS_2_, making MXenes attractive for applications such as flexible sensing, electromagnetic shielding, and energy storage.

In 2024, Gogotsi and collaborators reported a scalable fabrication strategy for MXene-coated fiber microelectrodes. Leveraging the high conductivity and processability of MXene dispersions, continuous coating of commercial nylon filaments at speeds up to 15 mm/s yielded conductive fibers with line resistance below 10 Ω/cm. These electrodes demonstrated excellent flexibility and mechanical robustness, maintaining performance even under knotting conditions. Their functionality was further validated in both in vivo and ex vivo applications, including neuromodulation, electrophysiological sensing, and H_2_O_2_ detection [76].

In the same year, Alex and co-workers developed MXene/PDMS/glycerol composite electrodes for wearable biosignal monitoring. Composite systems containing 15% and 20% MXene exhibited bulk impedances of 111 and 280 Ω and conductivities of 0.462 and 1.533 mS/cm, respectively, demonstrating promising sensing performance [89]. Furthermore, a 2024 study introduced a fabric-based lamina-emergent MXene electrode capable of maintaining stable skin-electrode contact under dynamic conditions. The resulting wireless electrophysiological monitoring platform successfully enabled gesture recognition using a convolutional neural network analysis [90] (Figure 3a).

A major challenge limiting MXene implementation is oxidative degradation under aqueous and oxygen-rich environments. Ti_3_C_2_T_x_ gradually oxidizes to TiO_2_ in aqueous systems, leading to a degradation of conductivity and structural deterioration. Current stabilization approaches include low-temperature storage, inert-atmosphere encapsulation, antioxidant incorporation, surface chemical modification, composite coating strategies, and the development of alternative MXene compositions with intrinsically improved oxidation resistance.

Beyond oxidative degradation, MXenes face additional engineering hurdles: dispersion shelf life is limited to weeks under ambient conditions [22,75]; ink formulation suffers from a narrow rheological window with high-concentration dispersions prone to gelation and restacking, causing nozzle clogging in printing processes [91,92]; flake-size distribution varies widely depending on etching conditions, affecting percolation thresholds and sensing consistency [75,76]; and batch-to-batch conductivity fluctuations of up to 1–2 orders of magnitude owing to precursor stoichiometry deviations and variable surface terminations remain unacceptable for industrial quality control.

### 2.3. Encapsulation Materials

The encapsulation layer is an indispensable component of flexible sensing electrodes, and its properties directly determine device environmental stability, biocompatibility, mechanical durability, and operational lifetime. An ideal encapsulation material must simultaneously satisfy several stringent requirements, including excellent water and oxygen barrier capability to protect sensing components from environmental degradation, high flexibility and stretchability to accommodate substrate deformation, favorable optical transparency, strong interfacial adhesion to prevent delamination, and application-specific biocompatibility or breathability. Accordingly, this section systematically discusses representative encapsulation materials from three categories: inorganic/organic thin-film encapsulation, polymer elastomer encapsulation, and emerging functional encapsulation strategies.

#### 2.3.1. Inorganic/Organic Thin-Film Encapsulation

Inorganic thin-film encapsulation primarily employs dense oxide and nitride films fabricated via atomic layer deposition (ALD) and CVD, such as Al_2_O_3_, SiO_2_, Si_3_N_4_, and TiO_2_. These materials exhibit extremely low permeability to moisture and oxygen, with the WVTR of Al_2_O_3_ on the order of 10^−6^ g/m^2^·day, thereby providing near-ideal environmental isolation for sensitive organic optoelectronic devices and implantable biomedical systems [93,94]. However, the inherent brittleness of inorganic thin films (fracture strain <1%) and their mechanical mismatch with deformable substrates often lead to crack formation and interfacial delamination under repeated mechanical stress. To address these limitations, alternating organic–inorganic multilayer architectures (e.g., Al_2_O_3_/polymer multilayer stacks) and nanoparticle-based toughening strategies are commonly employed to improve structural integrity and mechanical compliance.

Organic thin-film encapsulation generally relies on parylene-family materials and fluoropolymers. Among these, parylene C deposited via room-temperature CVD forms highly conformal coatings and exhibits exceptionally low moisture permeability, excellent dielectric properties, favorable biocompatibility, and compliance with established implant-grade safety standards (USP Class VI and ISO 10993 [95]). It can generate dense, pinhole-free coatings with highly uniform thickness (±5%) over complex 3D structures and is therefore widely employed to encapsulate implantable neural electrodes, pacemaker leads, and microfluidic systems [96].

#### 2.3.2. Polymer Elastomer Encapsulation

PDMS remains one of the most widely used polymer elastomer encapsulation materials because of its simple processing characteristics, excellent optical transparency, and compatibility with flexible electronics manufacturing. Surface plasma treatment can temporarily improve adhesion between PDMS and sensing layers by introducing polar functional groups; however, this enhancement is often transient due to hydrophobic recovery. Consequently, chemical grafting or mechanically interlocked architectures are frequently introduced to establish durable interfacial bonding [41,97]. Furthermore, the inherently high water vapor permeability of PDMS, which corresponds to relatively poor moisture-barrier capability, can compromise long-term wearing comfort and device reliability, and is therefore often mitigated through porous structural engineering or hydrophilic modification.

Styrene-based thermoplastic elastomers, including styrene-ethylene-butylene-styrene (SEBS) and styrene-isoprene-styrene (SIS), have attracted increasing interest for epidermal electronic encapsulation due to their excellent stretchability, thermoplastic processability, and favorable skin-adhesion characteristics [98]. In addition, PU and ultra-soft silicone elastomers such as Ecoflex are frequently used in applications requiring extremely high stretchability. Ecoflex exhibits elongation at break values approaching 900%, making it particularly suitable for motion-monitoring systems undergoing large mechanical deformation [28,99,100].

#### 2.3.3. Functionalized Encapsulation and Emerging Strategies

Beyond conventional protective and barrier functions, encapsulation layers are increasingly engineered with intelligent, multifunctional capabilities to meet the requirements of advanced wearable and implantable systems.

Self-healing encapsulation has emerged as an active research frontier in recent years [101,102]. Inspired by the intrinsic healing behavior of biological skin, dynamic covalent interactions, including Diels–Alder, disulfide, and borate ester bonds, as well as supramolecular interactions such as metal coordination and multiple hydrogen bonding, are incorporated into elastomeric networks. These dynamic interactions enable autonomous or stimulus-responsive repair under ambient conditions or mild heating after structural damage, thereby restoring both barrier integrity and mechanical performance. Self-healing encapsulation plays a critical role in enhancing device fault tolerance and extending operational lifespan, particularly for wearable electronics and soft robotic systems that are subjected to repeated deformation and mechanical stress [103,104].

Biomimetic skin-inspired encapsulation achieves synergistic optimization of breathability, waterproofing, and mechanical conformity by structurally and functionally imitating human skin. For example, encapsulation layers with gradient-modulus architectures effectively redistribute stress and suppress crack propagation, whereas skin-like microtextured surfaces can improve self-cleaning properties and strain-insensitive electrical characteristics, thereby enhancing signal fidelity during motion monitoring [105]. Moreover, skin-inspired sweat-regulating encapsulation structures have demonstrated significant benefits for improving long-term wear comfort [106].

Biodegradable encapsulation strategies address the requirement for noninvasive removal of implantable electronic systems. Encapsulation materials based on biodegradable polymers, including poly(lactic-co-glycolic acid) (PLGA), polycaprolactone (PCL), and silk fibroin, enable devices to gradually degrade and be absorbed in vivo after completing predefined therapeutic or diagnostic functions, thereby eliminating the need for secondary surgical removal [107]. Furthermore, degradation kinetics can be precisely regulated by controlling molecular weight, copolymer composition, and polymer crystallinity, thereby enabling adaptation to diverse therapeutic timelines and biomedical requirements [108].

### 2.4. Material Design Perspectives and Future Challenges

After years of development, flexible sensing electrodes have gradually evolved into a relatively established three-layer architecture comprising a flexible substrate, sensing layer, and encapsulation layer, with increasingly clear material selection strategies and design principles. Substrate materials have expanded from conventional elastomeric polymers to hydrogels and textile-based systems, enabling synergistic optimization of mechanical matching, breathability, and long-term wearing comfort. Significant progress has also been achieved in sensing materials, including AgNWs, liquid metals, carbon-based materials, conductive polymers, and MXenes, which have demonstrated remarkable advances in conductivity, stretchability, and processability. Meanwhile, encapsulation systems have evolved beyond passive protection toward multifunctional designs incorporating self-healing, biomimetic, and biodegradable features, thereby enhancing environmental robustness, mechanical durability, and biosafety.

From an application-matching perspective, material selection should be guided by target signal characteristics: PEDOT:PSS hydrogels are optimal for ECG/EMG owing to their mixed ionic–electronic conductivity and skin-matched modulus (kPa range) [16,84,85]; PI-based microneedle electrodes are preferred for EEG as they penetrate the stratum corneum, reducing interfacial impedance by >90% [13,43]; MXene/textile composites suit large-deformation motion monitoring (>200% stretchability) [18,90]; while carbon black/elastomer composites remain most viable for disposable low-cost devices [83,91].

Despite these advances, several critical challenges continue to hinder the scalable and long-term deployment of flexible sensing electrodes. Single-component materials often struggle to simultaneously satisfy multiple requirements, including high conductivity, large deformability, long-term operational stability, and biocompatibility. Representative examples include corrosion susceptibility of AgNWs, oxidation instability of liquid metals and MXenes, and weak interfacial adhesion on low-surface-energy substrates such as PDMS, which frequently causes delamination and limits device lifetime. Furthermore, the intrinsic tradeoff between breathability and barrier performance remains unresolved, while many emerging functionalities, such as self-healing and degradability, remain largely at the proof-of-concept stage and lack systematic performance evaluation.

Future developments should focus on designing multifunctional conductive composites and multiscale conductive networks by synergistically integrating 2D materials (e.g., graphene and MXenes), one-dimensional structures (e.g., AgNWs and CNTs), and conductive polymers to simultaneously improve conductivity, flexibility, and interfacial stability. Advanced interface engineering strategies, including protective coatings, alloying approaches, and surface functionalization, should be further explored to address oxidation and adhesion issues. Dynamic crosslinked networks may provide room-temperature self-healing capability, while gradient or phase-separated architectures could balance barrier performance, permeability, and mechanical compliance. For biodegradable systems, systematic studies on degradation kinetics and stimuli-responsive degradation mechanisms are still required. Overall, although substantial progress has been achieved in materials and fabrication approaches, significant gaps remain before realizing medical-grade long-term wearable and implantable systems. Future breakthroughs will likely rely on multiscale material design, multidisciplinary optimization, and fundamental understanding of failure mechanisms under realistic operating environments, ultimately enabling high-performance, reliable, low-cost, and biocompatible flexible sensing electrodes.

## 3. Fabrication Methods of Flexible Sensing Electrodes

Fabrication strategies are the critical bridge between material selection and the ultimate performance of flexible sensing electrodes. Their selection directly governs structural fidelity, electrical characteristics, mechanical reliability, and manufacturability. Table 2 systematically compares representative fabrication approaches in terms of processing resolution, material compatibility, and production efficiency.

### 3.1. Printing Technology

Owing to advantages including digital design capability, mask-free operation, high material utilization efficiency, and rapid prototyping, printing technologies have emerged as one of the most promising fabrication strategies for flexible electrode manufacturing.

#### 3.1.1. Screen Printing

**Figure 4:**
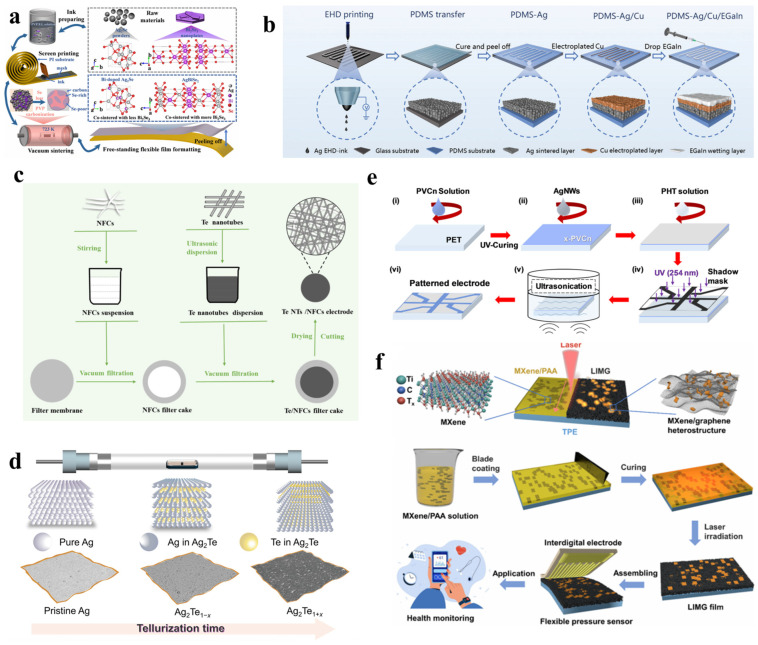
(**a**) Schematic of the fabrication of free-standing films via screen printing combined with co-sintering [131]. (**b**) Stepwise schematic of the m-SLE fabrication process: EHD printing of AgNPs, transfer to a PDMS substrate, electrodeposition of a copper layer, and selective wetting of EGaIn [115]. (**c**) Flowchart of the fabrication procedure for flexible Te electrodes [132]. (**d**) Schematic diagram of the preparation of Ag_2_Te thin films via PVD combined with Te vapor tellurization [120]. (**e**) Schematic illustration of the fabrication process of silver nanowire flexible electrodes by photolithography. Numbers (i)–(iv) represent the sequence of the process workflow [125]. (**f**) Schematic of the laser-induced conversion of MXene/polyamide acid (PAA) into a C–Ti–O bonded MXene/graphene heterostructure and the fabrication process of the MXene/graphene heterostructure (LIMG) flexible pressure sensor [133].

Screen printing is among the most mature and widely adopted fabrication methods in flexible electronics. This technique relies on applying pressure through a squeegee to transfer conductive ink through patterned meshes onto substrate surfaces. Its major advantages include operational simplicity, low equipment cost, broad material compatibility, and suitability for large-area and high-throughput production. Typical screen-printing resolution ranges from approximately 100–200 μm, while optimization of mesh density and emulsion thickness enables linewidths approaching 50 μm in high-precision systems. Conductive inks commonly consist of silver flakes, carbon pastes, or Ag/AgCl formulations, with viscosities generally ranging from 1000 to 10,000 cP [91,109]. A 2024 review by Li et al. systematically compared screen printing, inkjet printing, and 3D printing for ion-selective electrode fabrication and highlighted the excellent reproducibility and scalability of screen printing for preparing conductive contact layers, ion-to-electron transduction interfaces, and ion-selective membranes [92,110] (Figure 4a).

#### 3.1.2. Inkjet Printing

Inkjet printing deposits picoliter-scale droplets at predefined locations via piezoelectric or thermal bubble actuation, enabling non-contact, highly controllable patterned deposition. Major advantages of this technique include high design flexibility, exceptional material utilization efficiency (>90%), and compatibility with diverse flexible substrates. Commercial piezoelectric systems typically achieve printing resolutions of 20–50 μm with droplet volumes ranging from 1 to 10 pL [92]. One of the primary technical challenges associated with inkjet printing is optimizing conductive ink. Specifically, inks must simultaneously satisfy constraints on viscosity (typically 8–12 cP), surface tension (28–35 mN/m), and particle dispersion stability to ensure reliable droplet generation and high-quality film formation. Common optimization strategies involve the incorporation of surfactants, cosolvents, and polymer-based dispersants [111].

#### 3.1.3. EDH Printing and Other Advanced Printing Technologies

EHD printing employs high-voltage electric fields to extract ink from nozzles and generate ultrafine jets, thereby enabling patterning with submicrometer to several-micrometer resolution. Compared with conventional inkjet printing, EHD printing overcomes nozzle-size limitations and accommodates inks with broad viscosity windows ranging from 1 to 10,000 cP. Typical process parameters include applied voltages of 500–3000 V, nozzle-to-substrate distances of 0.5–2 mm, and feed rates of 0.1–5 μL/min. By maintaining stable Taylor cone formation, linewidths below 10 μm can be reproducibly achieved [112,113,114].

Ma et al. (2024) reported a PDMS/Ag/Cu/EGaIn microscale liquid-metal electrode (m-SLE) with linewidths of approximately 20 μm, stretchability approaching 100%, and mechanical durability exceeding 10,000 deformation cycles [115]. This system exhibited excellent mechanical robustness through an EHD-printed confined-template strategy, in which selective wetting directed liquid-metal self-assembly on electrodeposited Cu layers [77].

### 3.2. Solution Techniques

Solution-processing methods involve coating precursor solutions or dispersions onto substrate surfaces followed by solvent evaporation and post-treatment to form functional films, and they remain among the most widely used laboratory fabrication approaches. Spin coating employs centrifugal forces to distribute solutions uniformly across substrates, generating films with controllable thicknesses. Typical spin speeds range from 500 to 6000 rpm, enabling film thicknesses from tens of nanometers to several micrometers. In contrast, drop coating and dip coating involve simpler processing procedures and are suitable for the rapid preparation of small-area electrodes and 3D structures such as fibers and textiles. However, these methods typically exhibit film-thickness variation of approximately ±10–20% and poor batch reproducibility [115] (Figure 4b).

Vacuum filtration produces thin films by filtering conductive nanomaterial dispersions through microporous membranes with pore sizes generally ranging from 0.02 to 0.45 μm. This process is particularly suitable for 1D and 2D nanomaterials, including CNTs, graphene, and MXene, enabling accurate control of film thickness and areal density over the range of approximately 0.1–10 μg/cm^2^. The resulting films can subsequently be transferred onto arbitrary substrates, making vacuum filtration a commonly adopted approach for preparing highly conductive and transparent laboratory-scale films [116,117].

### 3.3. Vacuum Deposition and Coating Technologies

Physical vapor deposition (PVD) primarily includes thermal evaporation and magnetron sputtering. These methods vaporize source materials under vacuum conditions and subsequently deposit thin films onto target substrates. Thermal evaporation is suitable for low-melting-point metals, including Au, Ag, and Al, and typically exhibits deposition rates of 0.1–10 Å/s. Magnetron sputtering allows deposition of high-melting-point metals and oxide materials such as Pt and Ti with deposition rates reaching 10–100 nm/min. The resulting films generally exhibit high density, excellent adhesion, and superior electrical conductivity, making these techniques particularly suitable for high-precision microelectrode array fabrication [118,119,130] (Figure 4d).

CVD forms solid films through chemical reactions of gaseous precursors on substrate surfaces and has distinct advantages for the direct synthesis of graphene and CNTs. For example, monolayer graphene synthesized on Cu foil under CH_4_/H_2_ atmospheres at approximately 1000 °C exhibits high crystalline quality and excellent electrical characteristics, with carrier mobility exceeding 10^4^ cm^2^/V·s. These films can subsequently be transferred onto flexible substrates to fabricate transparent conductive electrodes. However, the high processing temperatures associated with CVD significantly restrict compatibility with most flexible polymer substrates [70,121,122,123].

### 3.4. Photolithography and Patterning Technology

Photolithography retains irreplaceable advantages in micro- and nanoscale electrode patterning due to its high resolution and mature industrial infrastructure. Conventional UV lithography achieves submicron resolution (typically 0.5–1.0 μm), whereas electron-beam and extreme-UV lithography can reach feature sizes on the order of several tens of nanometers or smaller. Nevertheless, traditional photolithography involves multiple process steps (including coating, exposure, development, etching, and resist stripping) and frequently requires harsh processing environments involving organic solvents, elevated temperatures, and plasma treatments, thereby imposing substantial challenges for flexible polymer substrates [125] (Figure 4e).

Nanoimprint lithography (NIL) transfers nanopatterns from templates into polymer layers via mechanical imprinting, achieving ultrahigh resolution (<10 nm) without requiring complex optical systems. Consequently, NIL exhibits considerable potential in flexible sensing electrodes, nanograting fabrication, and metamaterial architectures [124].

### 3.5. Laser Processing Technology

Laser processing has rapidly emerged as a powerful fabrication strategy for flexible electrode manufacturing because of its non-contact nature, mask-free operation, high precision, and programmable capability. In 2024, UV pulsed laser-assisted direct patterning of liquid-metal flexible devices was reported using nanosecond-pulsed 355 nm laser systems [78]. Furthermore, femtosecond laser-assisted, fishbone-inspired, selective liquid-metal dewetting approaches guide wetting behavior by constructing anisotropic low-surface-energy microstructures on flexible substrates, thereby generating residue-free liquid-metal patterns with sharp boundaries and high spatial resolution [79].

Laser-induced graphene (LIG) represents another breakthrough in laser fabrication technologies. LIG employs high-power laser irradiation of polymer substrates, such as polyimide, under ambient conditions, converting polymer surfaces into 3D porous graphene architectures via localized photothermal carbonization [126,127]. Zhao et al. (2025) developed an LIG@MXene composite for self-powered smart insoles that deliver output power densities of 35 V·cm^−2^ and monitor foot health via integrated triboelectric nanogenerators and sensors [128]. Su et al. (2024) reported femtosecond laser-induced MXene composite graphene exhibiting conductivity as high as 3187 S·m^−1^ for simultaneous biomarker detection and physiological monitoring [129]. Li et al. (2024) further demonstrated that MXene/graphene heterostructures exhibit a 567% increase in physiological sensing sensitivity compared with conventional LIG systems [133]. In a cost-reduction effort, Zhou et al. (2025) demonstrated a low-power CO_2_ laser direct-writing method processing polypropylene/Cu-org-glue/Cu composites in an ambient atmosphere without protective gas, with near-100% material utilization and ~6 W energy consumption [134], far below magnetron sputtering (~kW) [118,119] and CVD (~kW + high-temperature furnaces) [81,121].

### 3.6. Toward Scalable Fabrication of Flexible Sensing Electrodes

Current fabrication approaches for flexible sensing electrodes have evolved into a diversified technological framework. Printing technologies, including screen printing, inkjet printing, and EHD printing, have emerged as promising manufacturing strategies owing to their digitalized workflow, mask-free operation, and high material utilization efficiency. Solution-processing techniques, such as spin coating, drop casting, and vacuum filtration, offer simple and efficient routes for rapid preparation of nanomaterial-based films [135]. Vacuum deposition and CVD enable the fabrication of high-quality, dense films, whereas photolithography and NIL remain indispensable for micro- and nanoscale precision patterning. More recently, laser-assisted technologies, particularly LIG-based composite systems, have enabled noncontact and mask-free patterning with significantly improved manufacturing efficiency and sensing performance.

Despite these advances, substantial barriers remain toward large-scale manufacturing and commercialization. Screen printing suffers from limited patterning resolution (>100 μm), which restricts its applicability to microscale architectures. Inkjet printing requires stringent ink formulations and continues to face nozzle-clogging challenges. EHD printing involves complex parameter coupling among voltage, flow rate, temperature, and nozzle-to-substrate distance, making rapid standardization and large-scale reproducibility difficult. Solution-processing techniques frequently exhibit poor thickness uniformity and batch-to-batch consistency, with deviations of approximately ±15%, which limit manufacturing yield. Vacuum deposition and CVD require expensive infrastructure and often involve high-temperature processing (>300 °C), which is incompatible with many polymer substrates, including PDMS, PET, and PU. Conventional photolithography can damage mechanically compliant substrates, whereas laser processing remains constrained by precursor selectivity and limited large-area uniformity. For example, laser-induced carbonization strategies are currently effective primarily for selected precursors such as PI and remain difficult to apply directly to common substrates such as PET and PDMS.

To overcome these limitations, future technological development should focus on several directions. First, low-temperature and cost-effective patterning approaches tailored for flexible substrates, including transfer printing, soft lithography, microcontact printing, and roll-to-roll (R2R) continuous manufacturing, should be further explored. Second, optimization of printing ink rheology and interfacial chemistry is essential for improving process stability and expanding material compatibility. Multiscale collaborative fabrication strategies integrating different printing methods (e.g., inkjet-assisted EHD printing) may enable simultaneous improvements in patterning resolution and process versatility. Third, broader laser-carbonizable precursor systems and pretreatment strategies should be developed to improve material universality. In addition, integration of machine learning with automated process control may enable real-time parameter optimization and batch stability regulation, such as deep-learning-assisted tuning of EHD parameters or online quality monitoring of LIG fabrication. Finally, the development of R2R manufacturing technologies is critical for bridging the gap between laboratory-scale demonstrations and large-area, high-throughput production.

## 4. Practical Applications

### 4.1. Electrophysiological Signal Monitoring

Electrophysiological signal monitoring is one of the most important and extensively explored applications of flexible sensing electrodes and involves the acquisition of physiological bioelectrical signals, including ECG, EEG, electromyography (EMG), and electrooculography (EOG). Compared with conventional Ag/AgCl gel electrodes, flexible sensing electrodes offer distinct advantages in long-term wearability, mitigation of motion-induced artifacts, and stable dry-contact interfacing, making them highly promising platforms for continuous and high-fidelity physiological monitoring. In real-world deployment, however, motion artifacts remain the primary signal-quality compromiser—routine activities cause electrode-skin sliding and impedance fluctuations [1,2,3], and, while microstructured adhesives [43,136] and adaptive filtering algorithms offer partial mitigation, adhesion degradation and computational constraints persist. Additionally, prolonged wear (>24–48 h) can induce stratum corneum overhydration, pruritus, and irritant dermatitis [4,137], while certain conductive materials (Ag^+^ from AgNWs [66,67], Ga^3+^ from liquid metals [68,69]) pose a risk of cytotoxicity.

#### 4.1.1. ECG Monitoring

ECG signals are critical physiological indicators reflecting cardiac electrical activity and play indispensable roles in cardiovascular disease screening, postoperative rehabilitation, and daily health assessment. Conventional ECG systems generally employ disposable Ag/AgCl gel electrodes, which provide high signal quality but suffer from several intrinsic limitations, including gel dehydration (which typically restricts operation to <48 h), skin irritation, and susceptibility to motion artifacts, thereby limiting their suitability for long-term continuous monitoring.

In recent years, diverse flexible dry-electrode platforms have demonstrated superior ECG performance. One notable development involved a fiber-reinforced hybrid hydrogel (FRHH) electrode composed of PEDOT: PSS and Ti_3_C_2_T_x_ MXene. This electrode exhibited ultrahigh stretchability (~1485%) and an electrode-skin interfacial impedance of only 2829.3 Ω at 1000 Hz, substantially lower than those of commercial wet electrodes (6654.5 Ω) and conventional dry electrodes (17,611.2 Ω) [133]. Even under dynamic motion conditions, FRHH electrodes maintained stable conductivity and signal integrity, enabling continuous ECG and EMG acquisition [138]. Preliminary in vivo evaluations further demonstrated favorable biocompatibility, supporting continuous skin contact for up to 12 h without observable inflammation or allergic responses [137].

#### 4.1.2. EEG Monitoring

EEG records postsynaptic electrical activity in the cerebral cortex using scalp-mounted electrodes and is widely employed in epilepsy diagnosis, sleep analysis, brain–computer interfaces, and neuroscience research. Conventional EEG systems rely on conductive gels or pastes to reduce electrode-scalp impedance, a process that generally requires 30–60 min of preparation time. However, signal quality rapidly deteriorates as the conductive medium gradually dehydrates.

Flexible dry electrodes and microneedle-based electrodes have introduced transformative advances in EEG acquisition technologies. Microneedle electrodes penetrate the high-impedance stratum corneum and establish low-impedance electrical contact with deeper epidermal layers without requiring conductive gels, while simultaneously avoiding poor interfacial coupling commonly encountered in conventional dry electrodes. A flexible and mechanically robust microneedle electrode reported in 2024, based on nanowire-reinforced PI structures, combined high electrical conductivity with effective penetration performance. The microneedles accurately penetrated the stratum corneum without reaching dermal regions containing pain receptors, thereby enabling nearly painless and stable electrophysiological monitoring [43]. Other studies further integrated conductive textiles with microneedle architectures to develop conductive fabric-supported microneedle (CF-MN) electrodes capable of simultaneously supporting long-term, continuous, and high-quality ECG and EMG monitoring [136].

#### 4.1.3. EMG Monitoring and Human–Machine Interaction

Surface EMG (sEMG), which records muscle action potentials generated during contraction, is widely utilized in rehabilitation medicine, sports science, prosthetic control, and gesture recognition systems. High-density flexible electrode arrays can provide spatially resolved electromyographic information, enabling precise recognition of complex muscular activation patterns.

Several studies published in 2024 demonstrated innovative applications of flexible EMG electrodes in human–machine interaction systems. A PEDOT:PSS-modified flexible microneedle electrode array (P-FMNEA) was employed to construct a wireless facial biosensing platform for monitoring neuromuscular disorders such as facial paralysis through acquisition of facial EMG signals [130]. In clinical applications, a flexible sEMG system based on PEVA electrodes was evaluated for assessing lower back pain (LBP) and demonstrated favorable sensitivity and effectiveness in detecting electrophysiological indicators of lumbar dysfunction [139].

For wearable gesture-recognition applications, conductive textile-based MXene electrodes, combined with deformable structural designs, maintained a high signal-to-noise (S/N) ratio EMG acquisition under dynamic conditions and enabled highly accurate gesture classification through convolutional neural network analysis [90]. Similarly, FRHH hydrogel electrodes enabled precise EMG-based gesture recognition and analysis of muscle activation patterns [140].

### 4.2. Electronic Skin and Tactile Sensing

Electronic skin (e-skin) is a flexible sensing platform that emulates the tactile perception functions of human skin and has demonstrated considerable potential in intelligent robotics, prosthetic systems, and human–machine interfaces. Flexible electrodes in e-skin systems may function directly as sensing components for stimulus perception or as conductive transmission layers for signal collection and processing.

Dai et al. reported a bioinspired adhesive electronic skin incorporating a 3D structural architecture. Laser-induced adhesive interfaces enabled conformal contact with curved surfaces. Through a non-overlapping output strategy, the system achieved bidirectional differentiation of joint bending and decoupled sensing of strain and pressure stimuli. The device exhibited a pressure sensitivity of 0.652 kPa^−1^ within the 0–4 kPa range and a strain sensitivity factor of 8.13 at 0–15% strain. By establishing a ternary logic framework, the platform transformed mechanical stimuli into Morse code outputs and intelligent control commands, thereby demonstrating a functional pathway from physiological monitoring to human–machine communication [141].

### 4.3. Health Monitoring and Human–Machine Interaction Systems

Flexible sensing electrodes integrated into health-monitoring and human–machine interaction systems are gradually evolving from isolated sensing components to intelligent, multimodal, integrated platforms.

**Figure 5:**
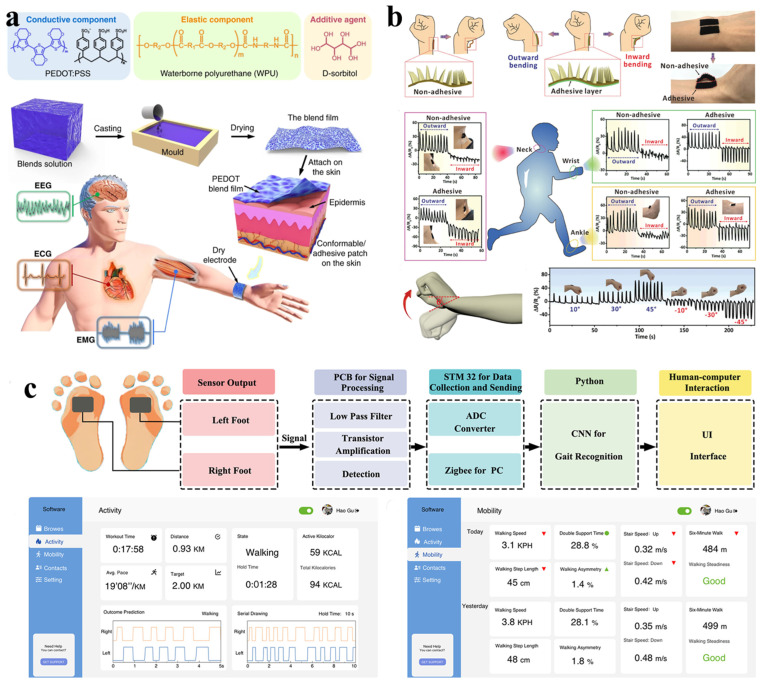
(**a**) Chemical structures of PEDOT:PSS, WPU, and D-sorbitol, the preparation process of the PWS hybrid film, and its application as an adhesive electrode for epidermal biopotential detection [5]. (**b**) Demonstration that non-adhesive electronic skin detaches from the skin during inward wrist bending, whereas the adhesive sensor enables stable monitoring of both inward and outward wrist bending (optical image comparison), and applies to multiple joints such as the neck and ankle; the resistance response varies with bending angle, showing different resistance values for inward bending at negative angles (−10° to −45°) and outward bending at positive angles (10° to 45°) [142]. (**c**) Human gait monitoring system: The device is fixed to the shoe sole, with data collected and uploaded, recognized via deep learning, and transmitted to a human–machine interface that includes “Activity” and “Mobility” interfaces [143].

In 2024, Gu et al. [142] developed an intelligent gait monitoring and prediction system that integrates flexible piezoelectric sensor arrays with deep learning neural networks. The sensor exhibited high sensitivity (241.29 mV/N), rapid response characteristics (66 ms loading and 87 ms recovery), and excellent reproducibility (R^2^ = 0.9946). When integrated into shoe insoles, the system enabled real-time acquisition and analysis of gait signals using customized deep learning algorithms, achieving gait recognition accuracy of up to 94.7%. This platform provides a promising technical strategy for personalized healthcare management, postoperative rehabilitation tracking, and early disease detection [143] (Figure 5c).

In the same year, multidirectional flexible strain sensors employing carbon/graphene composite sensing materials and optimized structural architectures demonstrated the capability to monitor subtle movements of the hand and neck, highlighting practical value in telemedicine and routine physiological assessment [142] (Figure 5b).

For low-cost electrochemical deployment, Li et al. (2025) developed a flexible potentiometric detector using commodity electronics, flexible PCBs, and screen-printed Ag/AgCl electrodes, achieving Na^+^/Ca^2+^ sensitivities comparable to commercial workstations with <4.3 mV deviation [144]. From a sustainability standpoint, the chitosan-based sensor by Amoah et al. (2025) offers a complementary solution: its >95 wt% biodegradable composition enables soil composting post-disposal, whereas an estimated 5 tons of non-recyclable PDMS waste would result from the annual production of one million conventional electrodes [59]. Together, these studies demonstrate that per-unit costs can be controlled within $20 while addressing end-of-life environmental impact, though challenges including ion-selective membrane drift (~9 mV/200 s) and long-term (>7 days) stability evaluation remain to be resolved [91,110].

## 5. Challenges and Prospects

Although flexible sensing electrodes have made substantial progress in materials innovation, fabrication technologies, and application expansion, multiple technological bottlenecks remain in the transition from laboratory-scale demonstrations to large-scale commercial implementation.

### 5.1. Long-Term Stability and Durability

Long-term operational stability remains one of the most critical challenges for flexible sensing electrodes. For liquid-metal systems, gallium-based materials readily form surface oxide layers under ambient conditions. Although these oxide layers can partially stabilize electrode morphology, challenges associated with dynamic wetting behavior, interfacial instability, and electrochemical corrosion remain unresolved. In MXene-based electrodes, oxidative degradation is a major limitation that affects long-term reliability. Ti_3_C_2_T_x_ gradually oxidizes to TiO_2_ in oxygen-rich aqueous environments, resulting in substantial deterioration in conductivity and structural integrity. In addition, fatigue-induced fracture of carbon-based conductive networks, under repeated deformation and irreversible doping/dedoping of conductive polymers during electrochemical cycling, can lead to signal drift and progressive performance degradation. Encapsulation strategies provide a critical pathway for improving operational stability. An ideal encapsulation layer should simultaneously exhibit low permeability to oxygen and moisture, high mechanical compliance, robust adhesion, and favorable biocompatibility. Although multilayer encapsulation architectures (e.g., Al_2_O_3_/polymer stacks) can achieve extremely low water and oxygen transmission rates, their mechanical incompatibility with flexible substrates often leads to microcrack formation and delamination under cyclic strain. Consequently, developing intrinsically stretchable and self-healing encapsulation systems with ultrahigh barrier performance remains a key research direction for addressing long-term stability challenges.

### 5.2. Scalable Manufacturing and Cost Control

Currently, the fabrication of most flexible sensing electrodes still relies heavily on laboratory-scale manual or semi-automated processing, which suffers from low production efficiency, poor batch consistency, and high manufacturing costs. Screen printing and inkjet printing technologies, owing to their compatibility with R2R continuous manufacturing, are considered among the most promising approaches for low-cost and scalable production. However, the transition from small-area laboratory fabrication (several cm^2^) to industrial-scale manufacturing (several m^2^) requires resolution of multiple engineering challenges, including maintaining conductive ink stability in large-volume production, controlling film-thickness uniformity over large areas (target deviation <±5%), ensuring accurate multilayer registration, and synchronizing drying behavior with high-speed printing processes (>10 m/min). In addition, large-area high-resolution liquid-metal patterning, long-term oxidation-resistant MXene inks, and universal fabrication strategies for LIG on non-PI substrates remain key technological barriers. The establishment of integrated online quality-control and feedback systems that enable intelligent, automated manufacturing processes is equally important for reducing production costs and improving production yield.

### 5.3. Biocompatibility and Skin Affinity

For flexible sensing electrodes that require prolonged skin contact or implantation, biocompatibility and tissue affinity are fundamental safety prerequisites. Hydrogel and textile substrates possess intrinsic advantages in this regard. The high water content of hydrogels helps maintain a hydrated interfacial microenvironment, thereby reducing skin irritation, while the breathable and moisture-wicking characteristics of textile materials minimize skin maceration and allergic responses associated with long-term wear. Preliminary in vivo studies demonstrated that FRHH hydrogel electrodes caused no inflammation or allergic reactions after 12 h of continuous skin contact, supporting the feasibility of this approach. For implantable systems, mitigation of the foreign body response (FBR) remains an important research priority. Following implantation, nonspecific protein adsorption, inflammatory-cell recruitment, and fibrous capsule formation can rapidly increase interfacial impedance, ultimately causing signal attenuation and device failure. Strategies including antifouling surface coatings (e.g., zwitterionic polymers and polyethylene glycol brushes), localized release of immunomodulatory agents, and ultra-soft material designs with moduli approaching the kilopascal range, comparable to brain tissue, are currently being explored. Furthermore, the establishment of comprehensive evaluation frameworks that address skin sensitization, cytotoxicity, and long-term implantation safety remains essential to facilitate clinical translation.

### 5.4. Multimodal Integration and Intelligence

Future flexible sensing platforms are expected to evolve toward multimodal perception, high-density integration, and intelligent operation. Single-modal sensing systems can provide only limited physiological information, whereas integration of multiple sensing modalities, including strain, temperature, humidity, and electrochemical sensing (e.g., sweat analysis), onto a single flexible platform may enable more comprehensive and accurate evaluation of physiological states. The deep-learning-assisted gait-analysis platform developed by Gu et al., which achieves 94.7% recognition accuracy, is a representative example of the synergistic integration of multimodal sensing and artificial intelligence, highlighting the significant potential of combining advanced sensing hardware with intelligent algorithms [143]. In addition, wireless communication capability and autonomous energy operation represent essential prerequisites for truly independent wearable systems. Wireless technologies, including near-field communication (NFC) and Bluetooth Low Energy (BLE), together with energy-harvesting approaches such as triboelectric nanogenerators (TENGs), flexible photovoltaics, and biofuel cells, are actively being explored to construct self-sustaining wireless sensing platforms. Future challenges will involve achieving high-density integration of multimodal sensing, signal processing, wireless communication, and energy management within constrained device dimensions while simultaneously maintaining low power consumption and minimizing signal interference during long-term operation.

### 5.5. Standardization and Market Prospects

The absence of unified performance evaluation criteria and standardized testing protocols remains a major obstacle to the industrial translation of flexible electrodes. Different research groups often report electrode performance under varying testing conditions (e.g., applied pressure range, strain rate, and environmental temperature and humidity), inconsistent definitions of evaluation metrics (e.g., sensitivity calculation methods), and different durations of long-term stability assessment, resulting in limited comparability and difficulty in establishing broadly accepted benchmarks. Therefore, developing industry-recognized standardized testing protocols and evaluation frameworks that encompass key parameters such as electrical conductivity, mechanical flexibility, interfacial impedance, biocompatibility, and accelerated environmental aging is essential to facilitate large-scale adoption and commercialization of flexible electrode technologies. Specifically, priority should be given to standardizing interfacial impedance measurement protocols (including frequency range, area normalization, and benchmark electrodes), mechanical cycling tests (requiring at least 1000 cycles at 0–50% strain with mandatory reporting of post-1000-cycle resistance drift), accelerated-aging protocols (40 ± 2 °C and 75 ± 5% RH, with retention reporting at 72 h, 7 days, and 30 days), and biocompatibility testing (ISO 10993-5 [145] and ISO 10993-10 [146], with 24 h and 7-day skin-reaction grading) [96,107].

## 6. Summary

Flexible sensing electrodes, as a key enabling technology for wearable health monitoring and human–machine interaction, have achieved remarkable advances in recent years across three major dimensions: material innovation, fabrication strategies, and application expansion. From a materials perspective, researchers have established a multilayer material framework comprising polymer elastomers, hydrogels, and textiles as substrate platforms; metal nanowires, liquid metals, carbon-based materials, conductive polymers, and MXenes as sensing components; and multilayer films, elastomeric matrices, and functionalized coatings as encapsulation systems. Significant progress has been achieved in integrating high conductivity, mechanical stretchability, self-healing capability, and biodegradability within multifunctional material systems. From a fabrication perspective, printing technologies, solution processing, vacuum deposition, photolithography, and laser-assisted manufacturing each provide distinct advantages. Among them, EDH printing and LIG technologies have created new opportunities for high-resolution, mask-free, and digitally programmable fabrication, whereas R2R continuous manufacturing offers a feasible pathway to low-cost, scalable production. From an application perspective, flexible electrodes have demonstrated performance advantages over conventional Ag/AgCl electrodes in electrophysiological monitoring applications, including ECG, EEG, and EMG, and have enabled emerging applications in electronic skin, intelligent gait analysis, and human–machine interaction systems.

Nevertheless, substantial gaps remain before large-scale commercialization and widespread clinical implementation can be achieved. Current challenges arise primarily from the inability of individual material systems to simultaneously satisfy multiple requirements, including high conductivity, large tensile deformation, long-term environmental stability, and excellent biocompatibility. In addition, superior laboratory-scale performance is frequently achieved at the expense of manufacturing scalability and cost-effectiveness. Furthermore, the absence of unified performance evaluation criteria and accelerated aging protocols limits cross-study comparability, impedes consensus development, and slows industrial translation.

Looking forward, flexible sensing electrodes are expected to continue evolving through the development of multifunctional composite materials, digitally integrated and modular fabrication approaches, system-level device architectures, and standardized evaluation frameworks. Through the convergence of materials science, manufacturing engineering, biomedicine, and information technology, this field is expected to transition from an academic research frontier to clinically relevant, consumer-oriented technologies over the coming decade, potentially transforming future healthcare systems and intelligent human–machine interactions.

## Figures and Tables

**Table 1:** Comparison of representative conductive materials for flexible sensing layers in terms of conductivity, stretchability, biocompatibility, environmental stability, and solution processability.

Material Category	Representative Material	Conductivity (S/cm)	Stretchability	Biocompatibility	Environmental Stability	Solution Processability	Refs
**Metal-based**	AgNWs	~10^5^ (individual nanowire); network value limited by junction resistance	Moderate; enabled by network sliding	Good	Prone to electrochemical corrosion and atmospheric sulfidation	Good (as printable ink)	[66,67]
**Metal-based**	Liquid Metal (EGaIn)	~3.4 × 10^4^ (bulk)	Excellent (>100% strain without conductive network failure)	Good (with encapsulation due to Ga^3+^ release)	Good, but forms an insulating oxide skin in air	Requires assisted patterning (e.g., EHD, laser)	[68,69]
**Carbon-based**	Graphene (CVD)	~10^3^–10^4^ (high-quality monolayer, doping-dependent)	Limited (intrinsically brittle); can be improved via serpentine or buckling structural design	Good	Excellent (chemically stable)	Poor; requires post-growth transfer to flexible substrates	[70]
**Carbon-based**	rGO	~10^0^–10^2^ (highly dependent on reduction method and defect density)	Limited	Good	Excellent	Excellent (processable as aqueous GO dispersion)	[71,72]
**Carbon-based**	CNT	~10^2^–10^4^ (network; depends on SWCNT/MWCNT ratio and alignment)	Moderate; facilitated by percolation network deformation	Good	Excellent	Moderate (requires surfactant or functionalization for stable dispersion)	[73,74]
**Conductive Polymer**	PEDOT:PSS	~10^−3^ to >10^3^ (highly tunable via secondary doping with organics or acids)	Moderate; can be enhanced by blending with plasticizers or soft polymers	Excellent	Moderate; hygroscopic, conductivity degrades in high humidity and under electrical bias	Excellent (commercial aqueous dispersion)	[22,29]
**MXene**	Ti_3_C_2_T_x_	~10^2^–2 × 10^2^ (≈ 10,000–20,000 S/m for pristine film)	Moderate; improved by compositing with elastomers (e.g., PDMS)	Good	Poor; prone to spontaneous oxidation into TiO_2_ in aqueous and oxygen-rich environments	Excellent (stable aqueous dispersion without surfactants)	[75,76]

**Table 2:** Comparison of fabrication methods for flexible sensing electrodes.

Fabrication Method	Resolution/ Thickness Control	Material Compatibility	Efficiency and Scalability	Typical Applications and Key Features	Refs
**Screen Printing**	50–200 μm (pattern)	Broad (high-viscosity conductive inks ~1000–10,000 cP)	High efficiency, R2R compatible	Large-area, low-cost biomedical electrodes; mature and reproducible	[91,109,110]
**Inkjet Printing**	20–100 μm (1–10 pL droplets)	Moderate (strict ink rheology: 8–12 cP, 28–35 mN/m)	Moderate, digital, and maskless	High-resolution microelectrode arrays, transistors, and high material utilization	[92,111]
**EHD Printing**	0.5–20 μm (sub-μm via Taylor cone)	Broad (wide viscosity window: 1–10,000 cP)	Low–moderate, high-precision, limited throughput	High-precision liquid metal/conductive polymer patterns; complex parameter coupling	[77,112,113,114]
**Spin/Drop Casting**	nm to μm (film thickness, ±10–20% variation)	Broad (solution-processable materials)	Low, batch process, poor consistency	Lab-scale thin-film preparation and device prototyping	[115]
**Vacuum Filtration**	10 nm to μm (film thickness, 0.1–10 μg/cm^2^ areal density)	Specific to 1D/2D nanomaterials (CNTs, graphene, MXene)	Low, batch process, requires film transfer	High-conductivity, transparent nanomaterial thin films	[116,117]
**Magnetron Sputtering**	Sub-nm control (10–100 nm/min)	Metals and oxides (high-melting-point source materials)	Moderate, wafer-scale deposition	High-precision, high-adhesion microelectrode arrays (PVD technique)	[118,119,120]
**CVD**	Sub-nm, monolayer control (~1000 °C, CH_4_/H_2_)	Specific to graphene, CNTs on catalytic substrates (e.g., Cu)	Low–moderate, high-temp. process, transfer-limited	High-quality, large-area monolayer graphene for transparent electrodes	[70,121,122,123]
**Photolithography**	0.1 μm–several μm (UV); <10 nm (NIL)	Resist-based (harsh solvents/plasma)	Moderate, multi-step, mature for rigid substrates	High-density microelectrode arrays for neural interfaces; NIL for nanogratings	[124,125]
**Laser Processing**	10–several μm (LIG linewidth; laser spot size)	Broad (PI carbonization to LIG, liquid metal patterning)	Moderate–high, maskless, direct-writing, programmable	LIG-based sensors and supercapacitors, liquid metal circuits; versatile and rapidly advancing	[78,79,126,127,128,129]
**3D Printing**	20–200 μm (nozzle/voxel size)	Photocurable resins/thermoplastics (often need post-coating)	Moderate, complex 3D geometries	Porous electrode scaffolds, structural substrates for bioelectronics	[17,18]

## Data Availability

No new data were created or analyzed in this study. Data sharing is not applicable to this article.

## Abbreviations

List of abbreviations used in this article with their full forms:

AAC	Acrylic acid
Ag/AgCl	Silver/silver chloride
AgNPs	Silver nanoparticles
AgNWs	Silver nanowires
ALD	Atomic layer deposition
BLE	Bluetooth Low Energy
CF-MN	Conductive fabric-supported microneedle
CNT	Carbon nanotube
CPI	Colorless polyimide
CVD	Chemical vapor deposition
ECG	Electrocardiography
EEG	Electroencephalography
EGaIn	Eutectic gallium–indium
EHD	Electrohydrodynamic (printing)
EMG	Electromyography
EOG	Electrooculography
FBR	Foreign body response
FRHH	Fiber-reinforced hybrid hydrogel
GO	Graphene oxide
LIG	Laser-induced graphene
LIMG	Laser-induced MXene/graphene
MXene	Two-dimensional transition metal carbide/nitride material family
MWCNT	Multi-walled carbon nanotube
NFC	Near-field communication
NIL	Nanoimprint lithography
PAA	Polyamide acid
PANI	Polyaniline
PCL	Polycaprolactone
PDMS	Polydimethylsiloxane
PEDOT:PSS	Poly(3,4-ethylenedioxythiophene):poly(styrenesulfonate)
PEEK	Polyether ether ketone
PEN	Polyethylene naphthalate
PET	Polyethylene terephthalate
PI	Polyimide
PLGA	Poly(lactic-co-glycolic acid)
PPy	Polypyrrole
PPSCF	PEDOT:PSS scaffold (hydrogel-related structure)
PPHG	PEDOT:PSS hydrogel variant
PU	Polyurethane
PVD	Physical vapor deposition
R2R	Roll-to-roll
rGO	Reduced graphene oxide
SEBS	Styrene-ethylene-butylene-styrene
SIS	Styrene-isoprene-styrene
SWCNT	Single-walled carbon nanotube
TATSAs	Triboelectric all-textile sensor arrays
TENGs	Triboelectric nanogenerators
T_g_	Glass transition temperature
TPU	Thermoplastic polyurethane
TPE	Thermoplastic elastomer
UV	Ultraviolet
WVTR	Water vapor transmission rate
